# A Meta-Analysis of In Vitro Release of Hydrophilic Therapeutics from Contact Lenses Using Mathematical Modeling

**DOI:** 10.3390/pharmaceutics17111479

**Published:** 2025-11-16

**Authors:** Lucia Carichino, Kara L. Maki, Narshini D. Gunputh, Chau-Minh Phan

**Affiliations:** 1 School of Mathematics and Statistics, Rochester Institute of Technology, 85 Lomb Memorial Drive, Rochester, NY 14623, USA; klmsma@rit.edu (K.L.M.); ng2447@g.rit.edu (N.D.G.); 2 Centre for Ocular Research & Education (CORE), School of Optometry & Vision Science, University of Waterloo, 200 University Avenue West, Waterloo, ON N2L 3G1, Canada; 3 Centre for Eye and Vision Research (CEVR), Unit 901-903, 17W Hong Kong Science Park, Shatin, Hong Kong

**Keywords:** therapeutic release, contact lens, diffusion coefficient, ophthalmic drug delivery

## Abstract

**Background/Objectives:** A meta-analysis was conducted to study the in vitro release of hydrophilic therapeutics from contact lenses, loaded using the soaking method. Fifty-three experiments were studied that measure the cumulative release of therapeutics from (mostly) commercial contact lenses placed in a vial. **Methods:** A mathematical model and a parameter-fitting algorithm are presented to estimate the diffusion coefficient (*D*) and 50% therapeutic release time ($T_{50}$) of all the experimental lens–therapeutic combinations. Statistical methods were used to analyze the relationships between lens materials, therapeutic properties, and predicted parameter values (*D* and $T_{50}$). **Results:** The mathematical framework was validated against previous studies. It was found that lens water content directly and moderately influences the estimated diffusion coefficient. More specifically, the median diffusivity of silicone hydrogel (SH) contact lenses was statistically different from that of conventional hydrogel (CH) lenses. The dependencies of other lens and therapeutic properties on diffusivity were complex, with special cases studied to elicit dependencies. A predictive tool was constructed to estimate the logarithm of 50% therapeutic release time ($\log(T_{50})$), given the lens water content and the therapeutic molecular volume and density. **Conclusions:** The conducted meta-analysis found that the kinetic release of therapeutics from contact lenses depends on the properties of both the contact lens and therapeutics. The statistical model explained 64% of the variability of the $\log(T_{50})$ and can be used in the preliminary stages of contact lens drug delivery development.

## 1. Introduction

Vision impairment, commonly caused by glaucoma, diabetic retinopathy, cataracts, and refractive error, affected 1.1 billion people worldwide in 2020, with the number expected to rise to 1.8 billion by 2050 [1]. Drugs for treating these diseases are usually administered by eye drops [2]. However, while eye drops are cost-effective and convenient, they suffer from low bioavailability due to spillage, dilution, blinking, and tear drainage after being administered to the eye [2,3]. The compliance rate of routine administration of eye-drop treatment is low [4]. One approach that has been proposed to improve drug delivery to the eye is the use of soft contact lenses [5]. These devices can not only act as a reservoir to provide sustained drug release but can also effectively shield the drugs from the various aforementioned ocular removal mechanisms [4,6]. Therefore, drug delivery via soft contact lenses can increase drug retention and bioavailability on the ocular surface [3,4]; among habitual contact lens wearers, treatment compliance is expected to improve [4].

Commercial contact lenses have continuously evolved, with the first soft contact lens approved by the FDA in 1971 [6,7]. The first manufactured soft contact lenses were conventional hydrogel (CH) contact lenses [6,7]. CH contact lenses differ in water content, as water content is important for on-eye performance and oxygen permeability, and ionicity [6,7]. Silicone hydrogel (SH) contact lenses, the next class of soft contact lenses launched in 1999, were introduced to increase oxygen permeability [6,7]. Subsequent generations of SH contact lenses have aimed to decrease the stiffness and increase the wettability of the lens caused by the additional silicone [6]. Commercial contact lenses are safe and easy to manufacture, making them a natural choice for the delivery of ophthalmic drugs [6].

Research on the use of contact lenses as a drug delivery vehicle has increased drastically in the past decade [4]. Acuvue Theravision, produced by Johnson & Johnson Vision Care, Inc. (Jacksonville, FL, USA), a ketotifen-releasing contact lens, was approved by the U.S. Food and Drug Administration (FDA) in 2022 [8]. However, issues with contact lens drug delivery remain, hindering widespread commercial availability of the technology [4]. For example, they include burst release within the first hours and the inability to maintain sustained therapeutic release over the desired treatment period, warranting further investigation [4,5,6,9]. The process of selecting a therapeutic and contact lens material is not yet optimized [4]. Additional challenges include therapeutic viability during manufacturing and preservation of contact lens properties after therapeutic loading [4].

Designing a contact lens to deliver a specific therapeutic at a specific dosage requires a predictable and controlled release. In vitro experiments evaluating drug delivery from contact lenses using a vial have shown that several factors can affect the drug release kinetics. SH lenses, which have a lower water content than CH lenses, tended to absorb fewer therapeutics than CH lenses and, consequently, release less [10]. Among SH lenses, those containing a higher portion of hydrophilic phases absorbed more therapeutics and released them faster [11]. For positively charged drugs, such as anti-muscarinic drugs atropine and pirenzepine, it was found that contact lens materials with a surface charge or ionicity released more drugs than non-ionic lenses [12]. Consequently, the exploitation of electrostatic interactions has been proposed to extend the therapeutic release duration of commercially available SH lenses [13]. The environment in which the lenses are placed also plays a role. For example, more sophisticated in vitro eye models with a low but constant fluid flow showed that the release of a therapeutic from a contact lens in that environment is much slower compared to a vial [14,15,16].

Mathematical models have been used to predict the release of a therapeutic from a contact lens and to improve lens delivery. Some models consider in vitro [17,18,19,20,21,22] release, while other consider in vivo release [23,24,25,26]. Theoretical studies focused on in vitro release have modeled the release of a loaded therapeutic from a contact lens via diffusion [13,19,20,21,22,23,24]. In general, these works estimate the diffusivity of a couple of lens–therapeutic combinations, assuming the dynamics of the drug carrier, i.e., the contact lens, can be ignored [27]. To the best of the authors’ knowledge, no previous study has been developed to explore how the predicted diffusivity changes with a wide range of lens and therapeutic properties. Additionally, no previous statistical tool has been developed to estimate the time-release kinetics of therapeutics from a contact lens in a vial given lens and therapeutics properties.

The aim of this study is to conduct a meta-analysis to characterize the dependence of lens and therapeutic properties on the release kinetics of hydrophilic therapeutics from pre-soaked contact lenses placed in a vial of fluid. Fifty-three experiments, taken from eleven publications, were analyzed using a mathematical and numerical framework to estimate the diffusion coefficient of the lens–therapeutic combination. A partial differential equations mathematical model was used to model the therapeutic release from the contact lens, assuming that the dynamics were governed by Fick’s law of diffusion and sink boundary conditions at the lens/fluid interface, similarly to prior works [18,19,20,21,22,23,28,29]. A parameter estimation algorithm was developed to estimate the diffusion coefficient, using the least squares method and the numerical solution of the mathematical model obtained via the method of lines and centered finite differences. A statistical framework was used to understand variations in the estimated diffusion coefficients depending on lens and therapeutic properties and their combinations. The statistical analysis was performed using the Shapiro–Wilk test for normality, a t-test and Mood’s median test for comparisons, and Kendall’s rank correlation coefficient. Using multiple linear regression, the results of the mathematical and statistical analyses were used to build a predictive tool that can be used to estimate in vitro therapeutic release effectiveness given lens and therapeutic properties. An understanding of therapeutic transport and a theoretical prediction of its effectiveness can help facilitate the development of better materials for ocular drug delivery.

## 2. Materials and Methods

**Figure 1:**
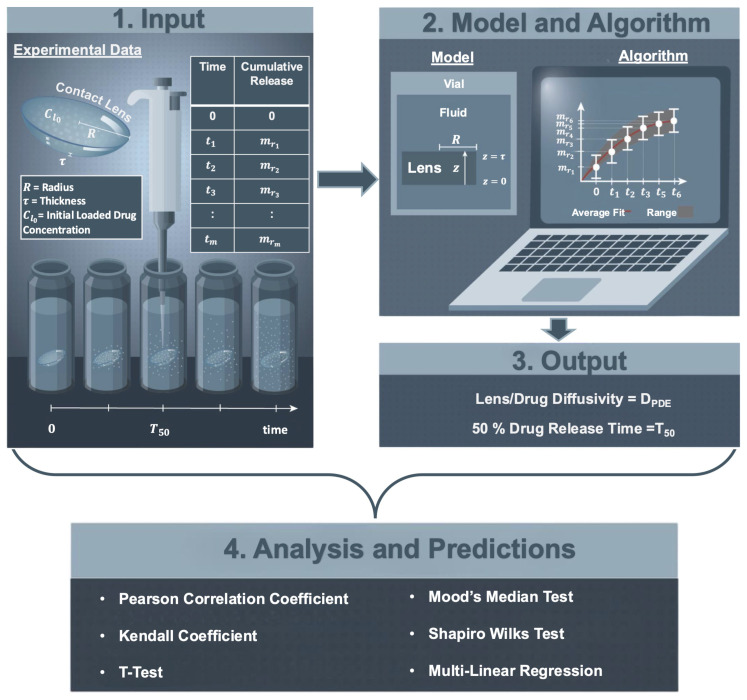
Schematic of the in vitro therapeutic delivery analysis. Starting from (**1**) experimental data, (**2**) a mathematical model and a fitting algorithm were used to estimate (**3**) the diffusivity and the release time of 50% of a therapeutic from a contact lens in a vial. (**4**) Contact lens and therapeutic properties, together with predicted diffusivities and 50% release times, were then analyzed using statistical methods. Modified version of an illustration created by Kerstyn Gay [30].

A schematic of the in vitro therapeutic delivery analysis is shown in Figure 1. Starting from the experimental data described in Section 2.1, a mathematical model is developed, together with a fitting algorithm, to estimate the diffusivity and the release time of 50% of a therapeutic from a contact lens in a vial. Section 2.2 explains the mathematical model used in the parameter estimation algorithm described in Section 2.3. Predicted diffusivities and release times were then analyzed in terms of lens and therapeutic properties using statistical methods as detailed in Section 2.4. Lastly, in Section 2.4, multiple linear regression is used to build a tool that can predict the timing of in vitro therapeutic release given lens and therapeutic properties.

### 2.1. Experimental Data

The fifty-three cumulative therapeutic release time series data for this study were taken from the eleven different publications listed in chronological order in Table 1. No new lab-based experiments were performed in this study. In vitro experiments considered measured the release of hydrophilic therapeutics from (mostly) commercial contact lenses placed in a vial. The therapeutic was loaded into the contact lens by soaking method. Note that, to the best of the authors’ knowledge, all the studies published before May 2024 were considered with hydrophilic therapeutics loaded in a contact lens via the soaking method and released in a vial. Studies that loaded more than one therapeutic to the lens, like a drug and vitamin E, were excluded from this work. In what follows, the general experimental procedure and the experimental data used for this study are explained in Section 2.1.1. Then, in Section 2.1.2, the partition coefficient estimates are presented. Finally, the different lens and therapeutic properties are characterized in Section 2.1.3.

#### 2.1.1. Cumulative Therapeutic Release Experiment

Studies referenced in Table 1 measured (in mass) the cumulative release of different therapeutics from SH and CH contact lenses after being placed in a vial of fluid (see Figure 1—1. Input). The in vitro therapeutic experiment had two parts: soaking and release in vials. First, a therapeutic was loaded into a contact lens by the soaking method. To do so, a contact lens was placed in a vial containing a reported therapeutic concentration, called the loading therapeutic concentration, denoted by $c_{v_{loading}}$, and reported in Appendix B Table A1, for a specified amount of time. Then, the therapeutic-loaded contact lens was placed in a vial of phosphate-buffered saline (PBS) with a volume of $v_{v}$, as reported in Appendix B Table A1. The amount of therapeutic released from the contact lens into the vial was measured over a twenty-four-hour period. This is the measured cumulative therapeutic release.

**Figure 2:**
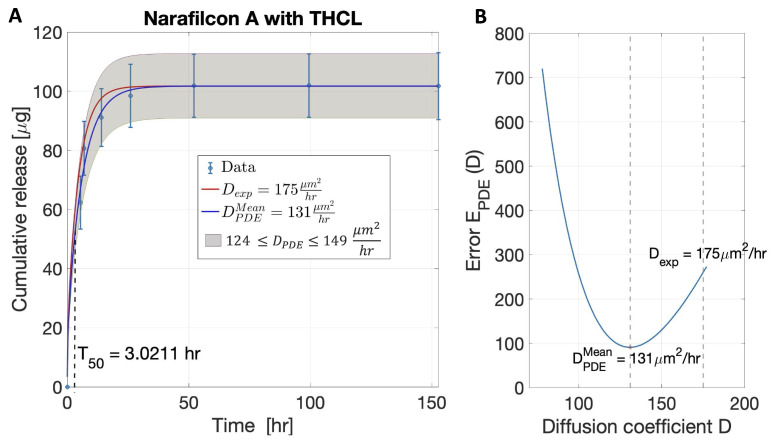
Parameter estimation for the release of tetracaine hydrochloride (THCL) from a narafilcon A contact lens. (**A**) The predicted cumulative therapeutic release ($m_{r}$) with $D=D_{exp}$ (red curve) and $D=D_{PDE}^{Mean}$ (blue curve). The shaded region is the predicted region of cumulative therapeutic release for the estimated range of diffusion coefficients. Data are presented as mean plus and minus the standard deviation. The dashed vertical line corresponds to the time that it takes for 50% of the loaded therapeutic to be released from the lens $\left(T_{50}\right)$. (**B**) The error ($E_{PDE}$) as a function of *D*.

For each lens–therapeutic pair, the experiment was run multiple times; consequently, the cumulative therapeutic release time-series datasets were reported as sample means with associated standard deviations. The experimental time-series are denoted by $\left(t_{1},m_{r_{1}}\right),\left(t_{2},m_{r_{2}}\right),$ …, $\left(t_{n},m_{r_{n}}\right)$, where $t_{i}$ is the time of the *i*-th measurement, $m_{r_{i}}$ is either the measured sample mean therapeutic mass in the vial or the measured sample mean mass plus and minus its sample’s standard derivation, and *n* is the total number of measurements. An example cumulative therapeutic release time-series dataset is shown in Figure 1—2. Model and Algorithm, where the circles denotes sample mean and the bars indicate plus or minus one standard deviation. Experimental datasets were either shared directly by the authors or were extracted from the publication.

#### 2.1.2. Estimated Partition Coefficient

The partition coefficient (*K*) is defined to be the ratio of the lens therapeutic concentration to the vial therapeutic concentration at equilibrium (end of soaking). The partition coefficient can be estimated by measuring the initial and final vial therapeutic concentrations during soaking [18]. However, for hydrophilic therapeutics, Dixon and Chauhan [18] found changes in the therapeutic concentration of the soaking solution could not be measured experimentally. Consequently, for experiments releasing hydrophilic therapeutics, Dixon and Chauhan [18] characterized the partition coefficient as follows:

(1) $$K=\frac{\left(\frac{m_{r_{n}}}{v_{l}}\right)}{c_{v_{loading}}},$$

where $m_{r_{n}}$ is the final measured therapeutic mass in the vial released from the contact lens, $v_{l}$ is the estimated lens volume, and $c_{v_{loading}}$ is the loading therapeutic concentration, that is, the therapeutic concentration in the lens at the end of soaking and was estimated by the released lens therapeutic concentration. Consequently, it was assumed that all the drug loaded into the contact lens was released in the subsequent experiment. Table A1 in Appendix B reports the estimated partition coefficients for all lens–therapeutic combinations considered in this work using Equation (1). Note that the values of *K* estimated using Equation (1) for senofilcon A and etaficoln A loaded with red dye (3.70 and 1.65, respectively) are of the same order of magnitude as the values of the partition coefficients estimated fitting the mathematical model developed by Anderson and Luke [24] to the same data (2.91 and 1.66, respectively; see Table 1 in [24]).

#### 2.1.3. Lens and Therapeutic Properties

A total of fourteen contact lenses were investigated in combination with different therapeutics, as described in Table 1. The key considered lens properties were lens material (SH vs. CH), FDA Group (Groups I–V) [33,34,35], ionicity [33,34,35], water content, thickness, (τ), and radius (*R*). FDA groups were defined as follows: (I) non-ionic, low-water-content (<50%) CH lenses; (II) non-ionic, high-water-content (>50%) CH lenses; (III) ionic, low-water-content CH lenses; (IV) ionic, high-water-content CH lenses; and (V) SH lenses [36]. In the considered datasets, SH lenses were classified as Group V and CH lenses as Groups II and IV, so no lenses from Group I and III were considered. A summary of the fourteen contact lens properties, organized by FDA group, is provided in Table 2. For each contact lens, the monomers are reported.

The properties of the examined therapeutics are reported in Table 3, including the molecular mass, density, molecular volume, and charge. The reported molecular mass and density were found in the literature, and molecular volume was computed by dividing the molecular mass by the density. Only three out of the fourteen considered therapeutics were negatively charged. Note that we do not include the target and/or eye disease treated by each therapeutic among the considered properties.

### 2.2. Mathematical Model

A mathematical model of therapeutic release from a contact lens was created. Two therapeutics concentrations were considered: the lens therapeutic concentration, denoted by $c_{l}$, and the vial therapeutic concentration, denoted by $c_{v}$. Variations in the lens therapeutic concentration were neglected in the radial direction, i.e., $c_{l}\left(z,t\right)$ was assumed to vary only along the thickness of the lens, represented by the *z* variable (see Figure 1—2. Model and Algorithm), and time (*t*). The contact lens was assumed to be loaded with a dilute, spatially independent concentration of therapeutic ($c_{l}\left(z,0\right)=c_{l_{0}}$) and placed into a vial with a volume of $v_{v}$ containing no therapeutic, that is, the vial therapeutic concentration ($c_{v}$) was zero at t=0, i.e., $c_{v}{|}_{t=0}=0$. When placed into the vial, the contact lens thickness was assumed to be constant, ignoring possible swelling dynamics and subsequent effects on drug transport [27].

At the lens surface, the lens therapeutic concentration was assumed to be proportional to the vial therapeutic concentration ($c_{l}=Kc_{v}$, where *K* is the partition coefficient defined in Equation (1)) [18,19]. Because the vial volume ($v_{v}$) was much larger than the lens volume ($v_{l}$), $c_{v}\approx 0$ was assumed as argued in Appendix A. Therefore, sink boundary conditions were assumed, that is,

(2) $$c_{l}\left(0,t\right)=c_{l}\left(\tau ,t\right)=0\;\mathrm{for}\;t>0.$$

The assumption of sink boundary conditions was also used in prior works [18,22,28,29].

The transport of the lens therapeutic was assumed to be governed by Fick’s law of diffusion [19,20,21,22,23]. Consequently, the lens therapeutic concentration ($c_{l}\left(z,t\right)$) was assumed to be governed by

(3) $$\begin{array}{c} \frac{\partial c_{l}}{\partial t}=D\frac{\partial^{2}c_{l}}{\partial z^{2}},\;0<z<\tau ,\;t>0,\\ c_{l}\left(0,t\right)=c_{l}\left(\tau ,t\right)=0,\;t>0,\\ c_{l}\left(z,0\right)=c_{l_{0}},\;0<z<\tau , \end{array}$$

where *D* is the diffusivity of the therapeutic in the contact lens. The analytical solution of Equation (3) was found by the method of separation of variables and is given by

(4) $$c_{l}\left(z,t\right)=\sum_{k=1}^{\infty }\frac{2}{\pi k}c_{l_{0}}\left(1-{\left(-1\right)}^{k}\right)\sin\left(\frac{\pi kz}{\tau }\right)e^{-\frac{D\pi^{2}k^{2}}{\tau^{2}}t}.$$

The cumulative release of the therapeutic from the contact lens was characterized by

(5) $$m_{r}\left(t;D\right)=v_{l}c_{l_{0}}-\pi R^{2}\int_{0}^{\tau }c_{l}\left(z,t\right)\;dz,$$

where $v_{l}=\pi R^{2}\tau$ and $c_{l}\left(z,t\right)$ is given by Equation (4). Note that $m_{r}\left(t;D\right)$ depends nonlinearly on the diffusion coefficient (*D*) via the exponential term in Equation (4).

### 2.3. Parameter Estimation Algorithm

A two-step algorithm was implemented to estimate the diffusion coefficient of the therapeutic from a contact lens from experimental cumulative therapeutic release time-series data.

**Inputs:** The inputs to the parameter estimate algorithm were

(i) An experimental time-series dataset of the cumulative release of the therapeutic from a contact lens placed in a vial of fluid, i.e., $\left(t_{1},m_{r_{1}}\right)$, $\left(t_{2},m_{r_{2}}\right)$, $\ldots ,\;(t_{n},m_{r_{n}})$;
(ii) The initial amount of therapeutic loaded into the contact lens, i.e., $c_{l_{0}}$;
(iii) The radius (*R*) and the thickness (τ), of the contact lens (see Figure 1—1. Input).

When total lens therapeutic absorption was provided, as reported by Torres-Luna et al. [13], the reported value was used to compute the initial condition ($c_{l_{0}}$); otherwise, $c_{l_{0}}$ was approximated using the average of the last three time points of the therapeutic cumulative release data, i.e.,
  (6) $$c_{l_{0}}=\frac{(m_{r_{n-2}}+m_{r_{n-1}}+m_{r_{n}})/3}{\tau \pi R^{2}}.$$

All contact lens radii (*R*) and thicknesses (τ) are reported in Table 2. All the values of the initial mass of therapeutics in the lens ($v_{l}c_{l_{0}}$) are reported in Appendix C Table A2.

**Step 1.** An approximation of the diffusion coefficient (*D*), denoted by $D_{exp}$, was found by fitting the experimental data to an exponential functional approximating Equation (5). More specifically, the time-series cumulative release data was fitted to an exponential function given by
  (7) $$m_{r_{exp}}\left(t;\alpha ,\beta ,\gamma \right)=\alpha +\beta e^{-\gamma t}+\delta e^{-9\gamma t},$$
  which was found by taking the first three terms of the series in Equation (4) and plugging them into Equation (5). The lsqnonlin nonlinear least squares solver in MATLAB 2022b (Natick, MA, USA) was used to find values of α, β, and γ that minimized the sum of the squares of the residuals given by
  (8) $$E_{exp}\left(\alpha ,\beta ,\gamma \right)=\sum_{i=1}^{n}{\left(m_{r_{i}}-m_{r_{exp}}\left(t_{i};\alpha ,\beta ,\gamma \right)\right)}^{2}.$$

The approximation of the diffusion coefficient ($D_{exp}$) was found by setting
  (9) $$D_{exp}=\frac{\gamma \tau^{2}}{\pi^{2}}.$$

**Step 2.** An estimate ($D_{PDE}$) was found by minimizing a different nonlinear least squares error given by
  (10) $$E_{PDE}\left(D\right)=\sum_{i=1}^{n}{\left(m_{r_{i}}-m_{r}\left(t_{i};D\right)\right)}^{2},$$
  where $m_{r}\left(t;D\right)$ was approximated using the numerical solution of the partial differential equation problem in Equation (3) and evaluating the integral of Equation (5). To find the numerical solution of Equation (3), the method of lines was used. The spatial partial derivatives were approximated by second-order centered finite differences ($\Delta z=\tau /N_{z}$, where $N_{z}=30$) and the temporal partial derivative by a forward finite difference ($\Delta t\;<\;\Delta z^{2}/D$). The integral in Equation (5) was approximated using the trapezoidal rule.

Starting from the value of $D_{exp}$, an interval of *D* containing $D_{exp}$ was identified (see Figure 2B), and the minimum of the error ($E_{PDE}$) was found inside such an interval. The algorithm was run until a minimum of Equation (10) was reached, with the corresponding diffusion coefficient denoted as $D_{PDE}$.

**Outputs.** Given the variability of the data, for each lens–therapeutic combination, a range of diffusion coefficients was estimated and a region (range) of cumulative therapeutic release was predicted. For each dataset, the algorithm was performed to fit the average data of cumulative release and obtain the average value of $D_{PDE}$, denoted as $D_{PDE}^{Mean}$. The average data plus or minus their standard deviations were fitted to obtain the upper and lower bounds for $D_{PDE}$.

The time at which the concentration of the therapeutic in the contact lens was equal to 50% of the initial amount loaded in the contact lens ($c_{l_{0}}$) was found, denoted as $T_{50}$. For each lens–therapeutic combination, the MATLAB find function was used to search for instances when $m_{r}\left(t;D_{PDE}^{Mean}\right)$ was within two decimal places of $\frac{v_{l}c_{l_{0}}}{2}$. The value of $T_{50}$ estimates the time that it takes for 50% of the loaded therapeutic to be released from the lens. The diffusivity ($D_{PDE}^{Mean}$) and time ($T_{50}$) depend on each other; in general, the larger the value of the diffusion coefficient, the faster the therapeutic will be released from the lens, i.e, the lower the value of $T_{50}$.

To measure the accuracy of the developed parameter estimation algorithm, we compute the average relative root mean square error ($\sqrt{E_{PDE}}/\left(v_{l}c_{l_{0}}n\right)$, where *n* is the total number of measurements) for all the considered lens–therapeutic combinations. The error is reported in the last column of Table A2 in Appendix C. For all the considered lens–therapeutic combinations, the error is less than 5% of the loaded therapeutic mass.

Figure 2A shows the algorithm-predicted cumulative therapeutic release of tetracaine hydrochloride (THCL) from a narafilcon A contact lens from Step 1 (red curve) and Step 2 (blue curve) and the experimental data (blue open circles) from Torres-Luna et al. [13]. The range of predicted $D_{PDE}$ is depicted by the gray region in Figure 2A. Figure 2B shows the error between the numerical solution and the data ($E_{PDE}$). The minimum of $E_{PDE}$ occurred at $D_{PDE}^{Mean}=131$ μm^2^/h, where the average relative root mean square error ($\sqrt{E_{PDE}}/\left(v_{l}c_{l_{0}}n\right)$) was less than 1% of the total amount of released therapeutic. The vertical dashed line in Figure 2A represents the predicted value of $T_{50}$ for the release of THCL from a narafilcon A contact lens.

### 2.4. Statistical Analysis

A total of eight continuous variables were considered: the diffusion coefficient ($D_{PDE}^{Mean}$) (found by fitting to the mean cumulative release data); the time to release 50% of the therapeutic from the contact lens ($T_{50}$); the water content, thickness (τ), and radius (*R*) of the contact lens; and the molecular volume, molecular mass, and density of the therapeutic.

Quantitative continuous variables were described by sample means, medians, and standard deviations (SDs). The normality of the sample data was first assessed using the Shapiro–Wilk test using the swft MATLAB function, with *p*-value > 0.05 and skewness between +1 and −1 [54]. Mean comparisons of the normally distributed data sampled independently from two unrelated groups were carried out with a *t*-test using the MATLAB ttest2 function. First, a two-tailed *t*-test was performed to assess the differences in means between the groups, and when the test was statistically significant, such differences were investigated further using an upper- or lower-tailed *t*-test. In general, a *p*-value < 0.05 was consider statistically significant. For non-normally distributed data sampled from two unrelated groups, Mood’s median test was carried out to assess the difference in their respective medians using the MATLAB mediatest function [55].

Since some of the variables were not normally distributed, Kendall’s rank correlation coefficient (*r*) was used to evaluate the correlation between continuous variables. The MATLAB corrplot function was used to calculate correlations.

Multiple linear regression analysis was performed to create a predictive tool for 50% therapeutic release times for a combination of lens and therapeutic properties. The MATLAB regress function was used. The normality of the residuals was verified using the Shapiro–Wilk test, with *p*-value > 0.05 and skewness between +1 and −1. Data whose corresponding residual did not follow a normal distribution were considered outliers and were removed, as detailed in Appendix D. The value of the coefficient of determination, denoted by $R^{2}$; the corresponding *p*-value; the value of the regression coefficients; and the corresponding 90% confidence intervals (CI) are reported.

## 3. Results

Validation of the parameter estimation algorithm is presented first in Section 3.1, followed by the meta-analyses of the estimated diffusion coefficients in Section 3.2. Then, a predictive tool is introduced to estimate release times of therapeutics from contact lenses in Section 3.3.

### 3.1. Validation of the Model and Algorithm

The algorithm was validated by comparing the estimated diffusion coefficients to those reported in the literature. Lanier et al. [19] developed a mathematical model to estimate the partition coefficient (*K*) and diffusion coefficient (*D*) of a contact lens during in vitro uptake and release of hydrophobic therapeutic cyclosporine. Diffusional transport of the therapeutic was assumed. The $\sqrt{D}K$ parameter was fit to the in vitro soaking data, and the *K* parameter was fit to the in vitro release data. In vitro experiments were conducted on five different lens types; then, parameters were estimated for each lens type. The results of the parameter estimation algorithm presented herein were compared to results of the HEMA-based lenses (one of the five different types), as the HEMA lenses’ cumulative release profiles were close to sink conditions (as discussed by Lanier et al. [19] and assumed here) and the HEMA monomer is also found in some of the commercial lenses considered in the work (see Table 2). Note that cyclosporine is a hydrophobic therapeutic; however, its cumulative release was approximated by Lanier et al. [19] assuming diffusion transport. For this reason, cyclosporine was included in the validation of the algorithm, but no other hydrophobic therapeutics were considered in the rest of the manuscript.

Table 4 reports the estimated diffusion coefficient ($D_{PDE}$), and the lens dimensions and the initial loaded theraputics mass used to estimate $D_{PDE}$. The assumed initial therapeutic mass loaded into the contact lens was estimated from the reported experimental load data (see Figure 4 of Lanier et al. [19]). Specifically, the difference between the first reported drug mass in the vial and the last reported mass is used; such a difference was assumed to be absorbed by the soaking lens. The estimated diffusion coefficient falls into the range of Lanier et al. [19]’s estimate. The error relative to the reported mean is 0.7%.

Pimenta et al. [20] developed a mathematical model to estimate the diffusion coefficient in single-layered and multi-layered systems. The release of levofloxacin and chlorhexidine from HEMA-based discs was considered. The model included the two parameters of *D* and α, which represent the diffusivity and the mass transfer between layers in the multi-layered lens system, respectively [20]. Pimenta et al. [20] estimated the *D* and α parameters by comparing the numerical solution of the mathematical model to the experimental solution, then adjusting them accordingly. When validating the model presented herein in comparison to that proposed by Pimenta et al. [20], only single-layered lenses and soluble drug levofloxacin were considered. Chlorhexidine was not considered, since it is insoluble.

Table 4 compares the predicted diffusion coefficient with the reported value of diffusivity for the combination of a single-layered HEMA contact lens with levofloxacin (see Table 1 in Pimenta et al. [20]). An initial mass of 24.52 mg of levofloxacin was assumed to be absorbed by the lens during the soaking process (same amount of levofloxacin released, under sink boundary conditions). The estimated diffusion coefficient of $D_{PDE}=2780$ μm^2^/h was close to the diffusivity of D=2700 μm^2^/h reported by Pimenta et al. [20]; the relative error is 3%.

Anderson and Luke [24] developed a mathematical model to study drug delivery from contact lenses in vivo. Their model accounts for diffusion of the drug out of the contact lens, transport into the pre-lens and post-lens tear film, and absorption into the ocular and eyelid tissue. Their model accounts for the partition coefficient (*K*) at the interface between the lens and the fluid. A simplified version of the model is used to fit the in vitro cumulative release of red dye from senofilcon A and etafilcon A lenses in a vial, as reported by Phan et al. [16], to estimate the the partition coefficient (K) and the diffusion coefficient (*D*).

Table 4 compares the predicted diffusion coefficient with the estimated diffusivity for the combinations of senofilcon A and etafilcon A lenses with red dye (see Table 1 as reported by Anderson and Luke [24]). The initial mass of red dye absorbed by the contact lenses was reported in [16] and in Table 4. The mean $D_{PDE}$ estimated in this work and the *D* prediction reported in [24] considered the average initial mass. Table 4 also reports the ranges of $D_{PDE}$, accounting for the variability in the data, as detailed in Section 2.3. The mean $D_{PDE}$ predicted in this work is of the same order of magnitude and 5–8% lower than the *D* predicted by Anderson and Luke [24]. The value of *D* in [24] for senofilcon A falls in the range of $D_{PDE}$ predicted in this work, while that for etafilcon A is close to the upper bound of the range predicted in this work.

### 3.2. Meta-Analysis of Estimated Diffusion Coefficients

**Figure 3:**
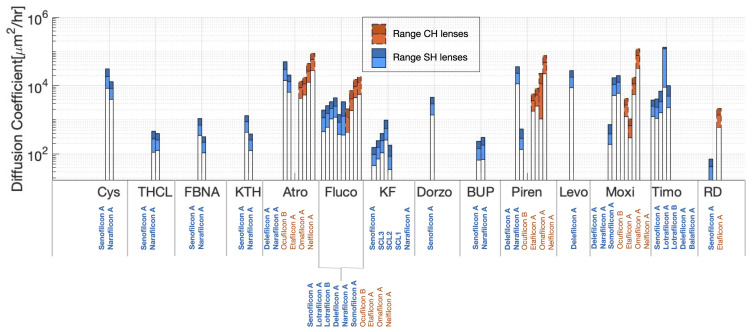
Predicted ranges of $D_{PDE}$ for all the considered combinations of contact lenses and therapeutics. The lenses were divided into commercial hydrogel (CH, orange names and dashed orange boxes) and silicone hydrogel (SH, bold blue names and solid-line blue boxes). The considered therapeutics were cysteamine (Cys), tetracaine hydrochloride (THCL), flurbiprofen sodium (FBNA), ketorolac tromethamine (KTH), atropine (Atro), fluconazole (Fluco), ketotifen fumarate (KF), dorzolamide (Dorzo), bupivacaine (BUP), pirenzepine (Piren), levofloxacin (Levo), moxifloxacin (Moxi), timolol (Timo), and red dye (RD).

Figure 3 shows the predicted ranges of $D_{PDE}$ for all considered combinations of contact lenses and therapeutics (see Table 1). The therapeutics, on the horizontal axis, are presented in order of ascending molecular mass (see Table 3). For each therapeutic, the lenses are divided in CH (orange names and dashed orange boxes) and SH (bold blue names and solid-line blue boxes) and, within each lens type, listed in order of ascending water content (see Table 2). The lighter colored bar represents the portion of the range of $D_{PDE}$ below $D_{PDE}^{Mean}$, and the darker colored bar represents the portion of the range above $D_{PDE}^{Mean}$. All the predicted values of $D_{PDE}^{Mean}$ and the range of $D_{PDE}$ are reported in Appendix C Table A2.

**Figure 4:**
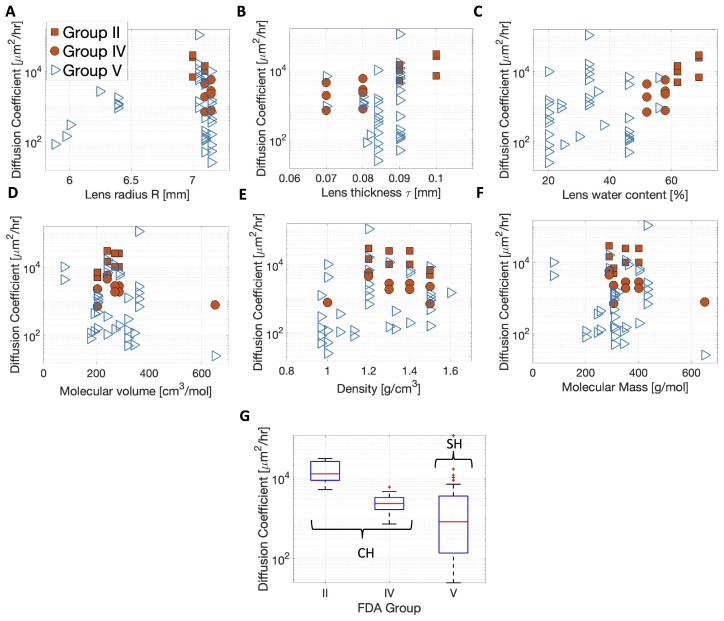
(**A**–**F**) The predicted values of $D_{PDE}^{Mean}$ shown on a logarithmic scale versus contact lens and therapeutic properties. (**G**) Boxplot of $D_{PDE}^{Mean}$ (shown on logarithmic scale) divided by FDA groups. In (**A**–**F**), silicon hydrogel (SH) lenses are reported in blue and commercial hydrogel (CH) lenses in orange. Different marker style symbols are used to represent the different FDA groups.

The predicted values of $D_{PDE}^{Mean}$, in logarithmic scale, are shown versus the contact lens and therapeutic properties in Figure 4. SH lenses are reported in blue and CH lenses in solid orange, and different symbols are used to identify the different FDA groups. Correlations were computed between the $\log(D_{PDE}^{Mean})$ and the lens and therapeutic properties considered in Table 2 and Table 3, and are shown in Table 5. Qualitatively, the predicted values of $D_{PDE}^{Mean}$, in logarithmic scale, were found to increase with water content (Figure 4C), and therapeutic density (Figure 4E). Kendall’s rank correlation analysis shows that $\log(D_{PDE}^{Mean})$ was positively correlated with water content (r=0.34, p<0.001) and the density of the therapeutic (r=0.23, p=0.020). No statistically significant correlation was found between $\log(D_{PDE}^{Mean})$ and the remaining lens and therapeutic features.

The correlation analysis reported in Table 5 shows that, in the dataset considered, radius and thickness of the contact lens are negatively correlated (r=−0.24, p=0.036). Additionally, therapeutic molecular volume is negatively correlated with therapeutic molecular density (r=−0.29, p=0.004) and positively correlated with the therapeutic molecular mass (r=−0.65, p<0.001). This is related to how the therapeutic molecular volume is computed, i.e., by dividing the molecular mass by the density.

FDA groups were defined based on differences in water content (Groups I and III have low water content, and Groups I and IV have high water content) and ionic charge (Groups I and III are non-ionic, and Groups III and IV are ionic). Figure 4G shows the boxplot of the predicted values of $D_{PDE}^{Mean}$ divided by FDA groups. The sample mean, median, and standard deviation of $D_{PDE}^{Mean}$ are reported in Table 6 for the CH lenses, SH lenses, and the corresponding FDA groups. The differences in $D_{PDE}^{Mean}$ were investigated statistically according to the type of lens, CH vs. SH, and FDA group. Samples from Groups II (N=8, Shapiro–Wilk test, p=0.204) and IV (N=9, Shapiro–Wilk test, p=0.333) were normally distributed, so t-test analysis results are reported in Table 6. CH lens (N=17, Shapiro–Wilk test, p=0.001) and SH lenses (N=36, Shapiro–Wilk’s test, p<0.001) (same as Group V) samples were not normally distributed, so Mood’s median analysis results are reported in Table 6. The median estimated diffusion coefficient of SH lenses was statistically different from that of the CH lenses (p<0.001). Group II, when compared to Group IV, showed a statistically higher $D_{PDE}^{Mean}$ (p=0.008). The median estimated diffusion coefficients for Groups IV and V were statistically different (p=0.008).

The differences in the estimated diffusion coefficient ($D_{PDE}^{Mean}$) were studied for ionic lenses (N=10), non-ionic lenses (N=38), and lenses with unknown ionicity (N=5), as reported in Table 7. Mood’s median test was used as the non-ionic lens samples (Shapiro-Wilk’s test, p<0.001), and samples of unknown ionicity (Shapiro-Wilk’s test, p=0.002) were not normally distributed. A statistically significant difference was found when comparing the median $D_{PDE}^{Mean}$ of ionic lenses with that of non-ionic lenses loaded with both positively and negatively charged therapeutics (p=0.033). When comparing ionic, non-ionic, and unknown-ionicity contact lenses loaded with only the positively charged therapeutics (see Table 3), no statistically significant differences were found in the median of $D_{PDE}^{Mean}$ for the different lens ionicity groups. Not enough data were available to perform the same analysis when lenses were loaded only with negatively charged therapeutics (see Table 3).

### 3.3. Predictions of Therapeutic Release Times

A predictive tool was constructed for the logarithm of the mean 50% release time ($\log(T_{50})$), given lens and therapeutic properties, using a multiple linear regression analysis.

Correlation analysis was used to decide which lens and therapeutics properties should be considered in the multiple linear regression. The $\log(T_{50})$ dataset, containing all lens–therapeutic combinations, is reported in Appendix C Table A2. Kendall’s rank correlation analysis shows that $\log(T_{50})$ was statistically significantly correlated with water content (r=−0.43, p<0.001) and therapeutic density (r=−0.36, p=0.003); see Table 5. Therefore, the explanatory variables used in the regression were the lens water content ($w_{c}$, where $w_{c}\;\in \;\left[0,100\right]$) and the therapeutic density ($t_{d}$, where the units are g/cm^3^). Note that $\log(T_{50})$ and $\log(D_{PDE}^{Mean})$ are naturally correlated with each other, since the value of $D_{PDE}^{Mean}$ is used to compute $T_{50}$ as described in Section 3.2. However, $\log(D_{PDE}^{Mean})$ is excluded from the regression model; otherwise, the model would have been inconsistent.

The used multiple linear regression model is given by

(11) $$\log\left(T_{50}\right)=a_{0}+a_{1}w_{c}+a_{2}t_{d}+a_{3}w_{c}t_{d}+\epsilon ,$$

where $a_{j}$, j=0,…,3 denote the regression coefficients and ϵ is the error term. When fitted to all lens–therapeutic combinations (N=53), the  model’s coefficient of determination was $R^{2}=0.43$, i.e., only approximately 43% of the variance in $\log(T_{50})$ is explained by lens water content and therapeutic density.

**Figure 5:**
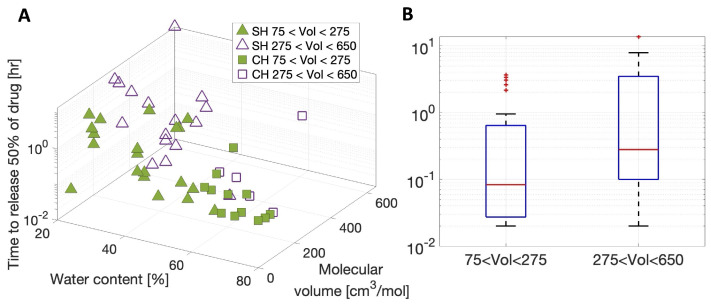
(**A**) Three-dimensional plot of $T_{50}$ (logarithmic scale) vs. lens water content and therapeutic molecular volume. The data are divided into two groups depending on the therapeutic molecular volume (Vol): volume between 75 ${\mathrm{cm}}^{3}/\mathrm{mol}$ and 275 ${\mathrm{cm}}^{3}/\mathrm{mol}$ (solid green) and volume between 275 ${\mathrm{cm}}^{3}/\mathrm{mol}$ and 650 ${\mathrm{cm}}^{3}/\mathrm{mol}$ (purple). Squares represent CH lenses, and triangles represent SH lenses. (**B**) Comparison of the box plot of the $T_{50}$ (logarithmic scale) for the considered therapeutic molecular volume groups.

The three-dimensional plot of $T_{50}$ (logarithmic scale) vs. lens water content and therapeutic molecular volume is shown in Figure 5A. The data are divided into two groups depending on the molecular volume: small molecular volume between 75 cm^3^ /mol and 275 cm^3^/mol (solid green, N=32) and large molecular volume between 275 cm^3^ /mol and 650 cm^3^/mol (purple, N=21). Squares represent CH lenses, and triangles represent SH lenses. Note that the two molecular volume ranges were chosen such that each of them includes both CH and SH lenses. Figure 5A shows that $\log(T_{50})$ qualitatively increases with water content and that such a trend might depend on the molecular volume of the therapeutic (different slope of the trend for the solid green symbols vs. purple symbols). Recall that molecular volume was computed by dividing the therapeutic’s molecular mass by its density.

Figure 5B compares the box plot of $T_{50}$ (logarithmic scale) in the two considered groups of molecular volume. Mood’s median test confirms the median $\log(T_{50})$ for the small-molecular-volume therapeutics was statistically different from the median $\log(T_{50})$ for large-molecular-volume therapeutics (p=0.0378). For these reasons, the multiple linear regression analysis was then performed on the small-molecular-volume therapeutics and the-large-molecular volume therapeutics separately.

**Figure 6:**
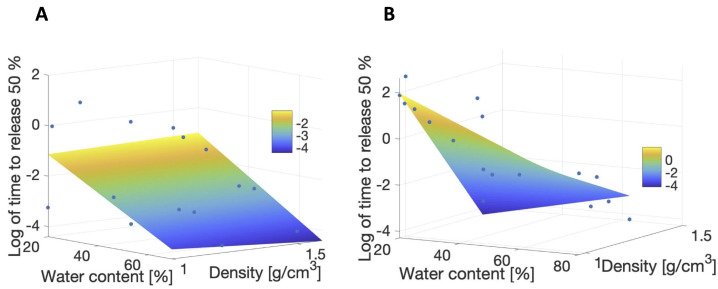
Graph of the predictive model for $\log(T_{50})$ (colored surface) vs. data (blue circles) for (**A**) small-molecular-volume therapeutics and (**B**) large-molecular-volume therapeutics.

A graphical representation of the theoretical predictive tool (surface plot) for small-molecular-volume therapeutics (A) and for large-molecular-volume therapeutics (B) is shown in Figure 6. The outliers (or data excluded to ensure normality of residuals), the coefficient of determination, $R^{2}$ with *p*-value, and the values of the regression coefficients ($a_{j}$) and their 90% confidence intervals (${\mathrm{CI}}_{j}$) are reported in Table 8. For more details on how the outliers were selected, we refer to Appendix D. The coefficient of determination ($R^{2}$) increased from 0.43 when considering all therapeutics together to 0.64 when considering only the small-molecular-volume therapeutics and 0.84 when considering only the large-molecular-volume therapeutics.

**Figure 7:**
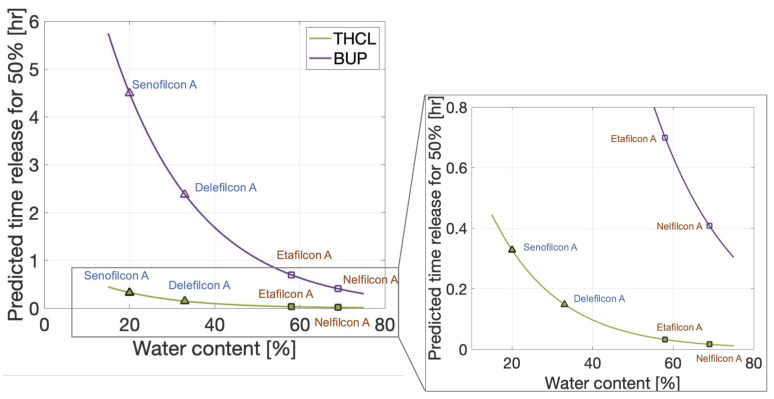
Predictive time of 50% release ($T_{50}$) of THCL (a small-molecular-volume therapeutic, green line) and BUP (a large-molecular-volume therapeutic, purple line) varying contact lens water content. Specific SH lenses senofilcon A and delefilcon A are denoted by triangle markers and blue labels, respectively, and CH lenses etafilcon A and nelfilcon A are denoted by square markers with orange labels.

Figure 7 shows the predicted 50% release time for small-molecular-volume therapeutic tetracaine hydrochloride (THCL, green line) and large-molecular-volume therapeutic bupivacaine (BUP, purple line) for varying contact lens water contents. The predicted time for 50% release is computed using Equation (11) with either the small-molecular-volume regression coefficients given in Table 8 and $t_{d}=1.13$ g/cm^3^ (THCL, Vol = 178.00 cm^3^/mol) or large-molecular-volume regression coefficients and $t_{d}=0.99$ g/cm^3^ (BUP, Vol = 343.07 cm^3^/mol). In general, the small-molecular-volume therapeutic was found to be released more quickly from contact lenses than the large-molecular-volume therapeutic. As the water content of the contact lens increased, the release time decreased.

**Figure 8:**
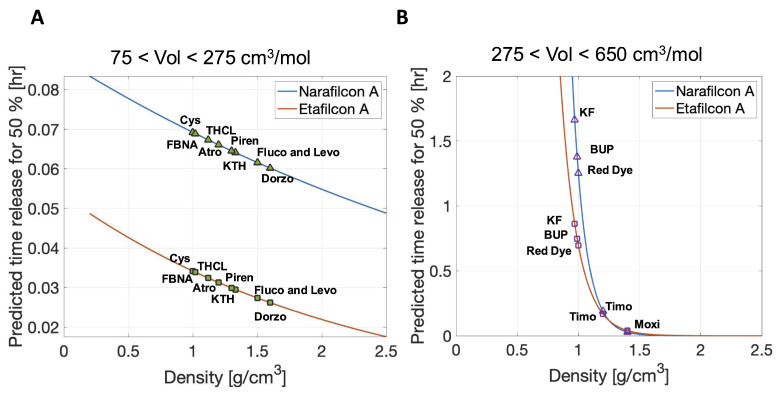
(**A**) Predictive 50% release time of small-molecular-volume therapeutics from SH contact lens narafilcon A (blue line) and CH contact lens etafilcon A (orange line) for varying therapeutic densities. Specifically shown small-molecular-volume therapeutics are tetracaine hydrochloride (THCL), flurbiprofen sodium (FBNA), ketrolomac tromethamine (KTH), atropine (Atro), fluconazole (Fluco), dorzolamide (Dorzo), levofloxacin (Levo), and pirenzepine (Piren). (**B**) Predictive 50% release time of large-molecular-volume therapeutics from SH contact lens narafilcon A (blue line) and CH contact lens etafilcon A (orange line) for varying therapeutic densities. Specifically shown large-molecular-volume therapeutics are ketotifen fumarate (KF), bupivacaine (BUP), moxifloxacin (Moxi), and timolol (Timo).

Similarly, Figure 8 shows the predictive tools applied to SH contact lens narafilcon A and CH contact lens etafilcon A for different therapeutic densities. Figure 8A shows the dependence of $T_{50}$ on therapeutic density for the narafilcon A lens ($w_{c}=46$, blue line) and etafilcon A lens ($w_{c}=58$, orange line) when considering small-molecular-volume therapeutics. The specific plotted therapeutics are tetracaine hydrochloride (THCL), flurbiprofen sodium (FBNA), ketrolomac tromethamine (KTH), atropine (Atro), fluconazole (Fluco), dorzolamide (Dorzo), levofloxacin (Levo), and pirenzepine (Piren). Figure 8B displays the predicted 50% release time of large-molecular-volume therapeutics for SH contact lens narafilcon A ($w_{c}=46$, blue line) and CH contact lens etafilcon A ($w_{c}=58$, orange line). The specifically shown therapeutics are ketotifen fumarate (KF), bupivacaine (BUP), moxifloxacin (Moxi), and timolol (Timo). Therapeutics, regardless of molecular volume, were released more quickly from an SH contact lens than from a CH contact lens. Small-molecular-volume therapeutics were released more quickly than large-molecular-volume therapeutics. As the density of the therapeutic increased, the therapeutic was released more quickly from the contact lens.

## 4. Discussion

The conducted meta-analysis, leveraging the mathematical framework developed herein (see Figure 1), found that the kinetic release of therapeutics from contact lenses depends on both contact lens properties and therapeutics properties. In particular, the timing of the release differed statistically significantly depending on the contact lens material (CH vs. SH), contact lens ionicity, and therapeutic molecular volume (i.e., molecular mass divided by the density), and it was correlated with the contact lens water content and therapeutic molecular density.

In agreement with previous works, the lens material is important in characterizing release kinetics [10,12]. In this work, therapeutics were found to be released faster, on average, from CH lenses than SH lenses. Table 6 reports that the median diffusivity of the CH lenses was statistically significantly different from the median diffusivity of the SH lenses, and Figure 5A shows CH lenses (squares) have shorter 50% release times than SH lenses (triangles). The timing of the therapeutic release was found to be one important piece of information when quantifying the dosage delivered to the ocular surface from a contact lens. Previous studies have reported that less therapeutics were released from an SH lens than a CH lens [10,12]. Consequently, therapeutics could be released more slowly from an SH lens, but the amount of therapeutic released could also be small.

Figure 3 shows that the predicted diffusivity associated with the delefilcon A lens appears to behave differently from other SH lenses for certain drugs. The delefilcon A lens had predicted diffusivities very different from those of other SH lenses when loaded with pirenzepine and moxifloxacin but like those of narafilcon A when loaded with atropine. The delefilcon A lens is marketed as a “hybrid” material (a hydrogel outer shell with a silicone core [6,12]). Thus, the likelihood of the release behavior of the hybrid lens mimicking other SH lenses depended on the therapeutic loaded into the lens.

The amount of water contained in the lens was found to correlate with therapeutic release timing. Specifically, high-water CH content lenses (Group II) were found to have statistically significantly greater mean diffusivity (smaller mean 50% release times) when compared to lower-water SH content lenses (Group V). In general, water content was found to be an important predictor of the therapeutic release time, regardless of whether the lens is a CH lens or an SH lens. Figure 5A shows the smaller predicted 50% release times for the lenses with high water content. A similar trend was found among the three experimental silicone-based lenses fabricated by Xu et al. [11] to study the release of ketotifen fumarate. The lens with the shortest release time of ketotifen fumarate had the highest measured equilibrium swelling ratio (a measure of the amount of water adsorbed by the contact lens). Additionally, the total amount of therapeutic loaded in and then released from a contact lens was found to depend on the water content of the lens [10,12,56,57]. Since therapeutics need to be solubilized in order to be released from the contact lens, in higher-water-content lenses, a higher amount of therapeutics can be solubilized and therefore released.

Thang et al. [58] argued that different levels of the composition and structures of hydrogels affect their swelling behavior, which, in turn, influences the overall kinetic time release of therapeutics. Chatterjee et al. [38] determined different compositions of contact lenses can affect the release of certain therapeutics from contact lenses. In general, SH lenses with TRIS monomers exhibited lower cross-linking, which, on average, led to a higher swelling capacity [38]. Therefore, the release of therapeutics from TRIS-containing SH lenses involved an initial burst release. In the studied datasets, balafilcon A, lotrafilcon A, lotrafilcon B, SCL1, SCL2, and SCL3 were the only SH lenses with TRIS monomers (see Table 2). For all combination therapeutics, the results showed that most SH lenses with TRIS monomers had a larger diffusion coefficient when compared to other SH lenses without TRIS monomers (excluding hybrid delefilcon A) such as narafilcon A and senofilcon A (see Figure 3).

Xu et al. [11] reported the different compositions of monomers in the SCL1, SCL2, and SCL3 experimental contact lenses with their corresponding different swelling ratios. SCL3 had the smallest diffusion coefficient and the lowest equilibrium swelling ratio, while SCL2 had the largest diffusion coefficient and the highest equilibrium swelling ratio. Thus, an increase in the diffusion coefficient and, consequently, faster therapeutic release, was predicted with an increase in swelling ratio, which is consistent with the results provided by Xu et al. [11]. Recall that, in this work, when placed into the vial, the contact lens thickness was assumed to be constant, ignoring possible swelling dynamics and subsequent effects on drug transport [27].

Among the high-water-content CH lenses (Groups II and IV), the non-ionic lenses (Group II) had a smaller median diffusivity than ionic lenses (Group IV) (see Table 7). Mood’s median test found the differences in the medians to be statistically significant. Consequently, the model predicted a slower median release of therapeutics from the non-ionic, high-water-content CH lenses than from ionic lenses. Prior works have found that surface-charged or ionic CH contact lenses affected the amount of therapeutics released; specifically, ionic lenses released more therapeutics [12]. Ionic contact lenses have a negatively charged surface that attracts positively charged therapeutics. For these reasons, it could be hypothesized that for some therapeutics, ionic, high-water-content CH lenses would release more therapeutics and do so more quickly than non-ionic, high-water-content CH lenses. When the therapeutics are absorbed by the contact lens, the hydrogel interacts with the adsorbed ions, inducing attractive or repulsive forces against the lens hydrogel structures [56]. In the future, to fully study how lens ionicity and therapeutic charge affect drug diffusion, the ionic permeability of the contact lens should be considered [59].

Etafilcon A, ocufilcon B, and balafilcon A were the only ionic contact lenses (Groups IV and V) studied herein. As shown in Figure 3, etafilcon A and ocufilcon B have the smallest diffusion coefficients among the subgroup of CH lenses when combined with positively charged drugs such as atropine, pirenzepine, and moxifloxacin. The negatively charged contact lenses might form electrostatic bonds with the cationic drugs, causing a slower release of the drug, which was captured in this work by smaller diffusion coefficients [60]. Balafilcon A is the only ionic SH lens investigated. Saez-Martinez et al. [61] investigated the microstructure of SH contact lenses and found balafilcon A to be the most ion-permeable, followed by lotrafilcon A and narafilcon A. Peng and Chauhan [62] also argued that ion permeability could influence the transport of ionic drugs in SH contact lenses such that lenses with higher ionic permeability would have larger diffusivity. In Figure 3, a similar trend is observed for combination contact lenses with timolol released, where balafilcon A had a larger diffusion coefficient, indicating faster release, which could be hypothesized due to its large ionic permeability.

In this work, statistical differences were found in the median diffusivity of therapeutics considered in the ionic lenses (N=10) and non-ionic lenses (N=38), but no statistical significance in the median diffusivity of the therapeutics was observed when comparing either the ionic or non-ionic lenses with unknown-ionicity contact lenses (N=5). Soluri et al. [63] found that contact lenses such as etafilcon A, etafilcon B, and balafilcon A, which are generally ionic contact lenses, had a larger uptake and release of therapeutics when compared to the non-ionic contact lenses, which supports the differences observed in the median diffusivities of ionic and non-ionic lenses. No statistical difference was found between the different ionicity lens groups when considering only positively charged therapeutics. Since only three out of the fourteen considered therapeutics were negatively charged (see Table 3), the same analysis was not performed considering only negatively charged therapeutics. For these reasons, lens ionicity and drug charge were excluded from the multiple linear regression model built in Section 3.3. In the future, if more data are available on the effect of the combinations of lens ionicity and drug charge on therapeutic release kinetics, it would be interesting to extend the regression model proposed in this work to account for their effect.

The partition coefficient (*K*) of each lens–therapeutic combination was estimated using Equation (1), with values reported in Table A1. The mathematical model used to estimate the cumulative therapeutic release in this work assumed a zero partition coefficient. The average relative errors of the estimated cumulative therapeutic release are reported in Table A2. The average relative errors were found to be weakly correlated with the estimated partition coefficient (r=0.25, p=0.009). In this study, the combination of delefilcon A with atropine had the smallest estimated partition coefficient, while narafilcon A with ketotifen fumerate had the largest partition coefficient. In comparison, as shown in Figure 3, delefilcon A with atropine has one of the largest diffusion coefficients (fastest release of atropine) compared to narafilcon A with ketotifen, which had one of the smallest diffusion coefficients (slowest release of ketotifen). On average, CH lenses had smaller partition coefficients in comparison to SH lenses, with the exception of delefilcon A (the “hybrid” lens).

Based on the experiments evaluated in this study, the molecular mass and the molecular volume (molecular mass/density) of the therapeutics were found to be not statistically correlated with diffusivity and therapeutic release time (see Table 5). Interestingly, diffusivity and therapeutic release time were found to be correlated with molecular density instead. However, therapeutic release time was statistically different between small- and large-molecular-volume therapeutics (see Figure 5). For these reasons, multiple linear regression analysis, expressing therapeutic release time as a function of molecular density and lens water content, was then performed separately for small- and large-molecular-volume therapeutics. For the large-molecular-volume therapeutics, the statistical model predicting the 50% release time described in Equation (11) had an $R^{2}$ value of 0.84 (see Table 8), i.e., capturing 84% of the variability of the data. The $R^{2}$ value was 0.64 for the small-molecular-volume therapeutics. Small-molecular-volume therapeutics were found to release faster than large-molecular-volume therapeutics. Karlgard et al. [10] reported similar trends, finding the smaller therapeutics to rapidly release from the contact lenses. Similar results were found by Hui et al. [12] when comparing the amounts of atropine and pirenzepine released to the amount of dexamethasone released from the same lenses.

There are limitations to the present work. Simplifying assumptions were used to create the mathematical model presented herein—most notably, the zero therapeutic concentration in the surrounding vial fluid, ignoring vial fluid dynamics, and the possible effects of contact lens swelling on drug release [27,56]. Prior works have quantified therapeutic concentrations in both the lens and surrounding fluid, in the appropriate limits, predicted diffusivities are similar [19,20,24]. The results of this study only pertain to in vitro therapeutic release kinetics through contact lenses loaded passively via the soaking method. In order to extend this work to predict therapeutic release kinetics from contact lenses in vivo, future work will tailor the mathematical model to account for eye blinking and tear film fluid dynamics and their interactions with pharmaceutic kinetics, similar to [24]. Additional techniques have been studied to load therapeutics in contact lenses, other than the soaking method, like molecular imprinting, nanoparticle-loaded contact lenses, and semi-circular acrylate ring-implanted contact lenses [64,65]. The results of this study cannot be extended to these additional loading techniques without modifying the mathematical model used to capture the key kinetic mechanisms associated with the specifically considered loading technique. This will be the focus of future work.

The predicted model of the 50% release time for a given therapeutic–lens combination, given in Equation (11), is a first step toward building a digital twin of in vivo hydrophilic therapeutic release from contact lenses. It can be used in the preliminary stages of contact lens therapeutic delivery development to predict the 50% release time for a given therapeutic–lens combination that has not been tested in the lab yet. Additionally, given a therapeutic and a target 50% release time, it can be used to predict the lens water content needed to reach such a target release time. The duration of the therapeutic release can be estimated; however, the amount of therapeutic released from the lens cannot be estimated, as it requires additional information on the amount of therapeutics loaded. Additionally, the predicted model cannot quantify therapeutic loss. Therefore, the initial amount of therapeutic loaded into the lens was assumed to be equal to the measured amount of therapeutic released from the lens.

In the multiple linear regression model, we use the iterative process detailed in Appendix D to remove outliers. This process is based on removing the data points corresponding to regression residuals that have a longer distance to the normal probability plot line. In the future, it would be interesting to explore if the method used to remove outliers has any effect on the predicted regression coefficients. A possible alternative to compare to the current model is using a regression loss function that reduces the effect of the outliers by reducing their weights, like the Huber loss function.

Among the eleven experimental studies considered, two studies reported the exact temperature when the experiments were conducted (37 °C [11] and 34 °C [12]), four reported that the studies were conducted at room temperature without reporting a temperature value [13,16,29,32], and the others did not report a temperature. Three of the eleven studies mentioned that the vial was shaken during the experiment [11,12,16]. For each contact lens, the monomers are reported in Table 2. However, information on contact lens composition, i.e., what percentage of the lens was made of which monomer, was not found in the literature. The therapeutics considered in this study (see Table 3) are used to treat a variety of ocular diseases, from allergic conjunctivitis to glaucoma, and only three of them are negatively changed. Due to the limited size of the available data sample, no analysis was performed to study the effects of the experimental design, like temperature and shaking vs. no shaking of the vial, the contact lens composition; the therapeutic target; and/or the treated eye disease. Additionally, correlations between lens ionicity and negatively charged therapeutics were not explored in this work. If more data were available in the future on these factors, it would be interesting to explore their effects on the results of this study by either standardizing the diffusivity predictions by these factors and/or performing a meta-regression, including them as variables.

Additionally, if all individual experimental data were available rather than only the reported mean and standard deviations, the current statistical model developed in Equation (11) could be extended to give a more robust interval estimate (rather than one value, as in this study) of 50% release time for a given therapeutic/lens combination via resampling methods such as bootstrapping. Additionally, more data will allow for a split into a training set and a test set and for a cross-validation to be performed to assess the predictive power of the proposed tool and to create a feedback loop between the data and the predictive tool to build a digital twin of therapeutic release kinetics in vitro.

## 5. Conclusions

A mathematical and statistical framework has been proposed herein to conduct a meta-analysis of the release of therapeutics from pre-soaked contact lenses placed in a vial of fluid. We analyzed fifty-three datasets obtained from the eleven publications detailed in Table 1, testing different combinations of fourteen contact lenses and fourteen therapeutics. The mathematical framework was based on the modeling of therapeutic release via diffusion with sink boundary conditions. Using the experimental data, the diffusivity and corresponding time for 50% of the therapeutics to be released were extracted and analyzed with respect to lens and therapeutic properties.

The water content of the lens was found to be positively correlated with the estimated diffusivity for all studied lens–therapeutic combinations. The median SH lenses released therapeutics differently than the median CH lenses. Consequently, the release time was hypothesized to be longer if a therapeutic was released from an SH lens when compared to a CH lens. Additionally, the mean Group II CH lens released therapeutics more quickly than the Group IV CH lens for the studied lens–therapeutic combinations.

The dependence of therapeutic release given the therapeutic charge and lens ionicity was found to be more nuanced, depending on whether only positively charged therapeutics or only high-water-content lenses were considered. When considering all therapeutics (both positively and negatively charged), the non-ionic lenses had a statistically different median diffusivity than ionic lenses. Statistically significant differences in medians were not found when only considering positively charged therapeutics. Not enough data was available to study negatively charged therapeutics individually.

Finally, it was found that the 50% release times of therapeutics were statistically significantly correlated with lens water content and therapeutic density. Interestingly, therapeutic molecular volume, therapeutic density, and contact lens water content could explain at least 64% of the variability in predicted 50% release times of therapeutics using multiple linear regression. The predicted model of the 50% release time for a given lens–therapeutic combination, given in Equation (11), can be used in the preliminary stages of contact lens drug delivery development to estimate the release time.

## Figures and Tables

**Table 1:** List of publications from which the datasets were extracted, with details on the considered contact lenses and therapeutics. For the first publication, the balaficon A contact lens is only loaded with timolol.

Reference	Therapeutics	Lenses
Peng et al. [21]	Fluconazole Timolol	Balafilcon A
		Lotrafilcon A
		Lotrafilcon B
		Senofilcon A
Xu et al. [11]	Ketotifen fumarate	SCL1 SCL2 SCL3
Bajgrowicz et al. [14]	Moxifloxacin	Delefilcon A
		Etafilcon A
		Narafilcon A
		Nelfilcon A
		Ocufilcon B
		Omafilcon A
		Somofilcon A
Hsu et al. [31]	Dorzolamide	Senofilcon A
Phan et al. [15]	Fluconazole	Delefilcon A Etafilcon A Narafilcon A Nelfilcon A Ocufilcon B Omafilcon A Somofilcon A
Dixon and Chauhan [18]	Levofloxacin Timolol	Delefilcon A
Hui et al. [12]	Atropine Pirenzepine	Delefilcon A Etafilcon A Narafilcon A Nelfilcon A Ocufilcon B Omafilcon A
Dixon et al. [29]	Cysteamine	Narafilcon A Senofilcon A
Torres-Luna et al. [32]	Flurbiprofen sodium Ketorolac tromethamine	Narafilcon A Senofilcon A
Torres-Luna et al. [13]	Bupivacaine Ketotifen fumarate Tetracaine hydrochloride	Narafilcon A Senofilcon A
Phan et al. [16]	Red dye	Etafilcon A Senofilcon A

**Table 2:** Description of studied contact lenses and their properties. Abbreviations are as follows: EGDMA: ethylene glycol dimethacrylate; FMA: N-Formylmethyl acrylamide; PVP: polyvinylpyrrolidone; NVP: N-vinylpyrrolidone; DMA: N,N-Dimethylacrylamide; PC: phosphorylcholine; MPDMS: monofunctional polydimethylsiloxane; TEGDMA: tetraethleneglycol dimethacrylate; PVA: polyvinyl alcohol; HEMA: hydroxyethyl methacrylate; MAA: methacrylic acid; TRIS: 3-(trimethylsiloxy)silylpropyl methacrylate; PEG: polyethylene glycol; FMA: fluorinated methacrylate; MA-PDMS-MA: polydimethylsiloxane (PDMS) long chains with methacrylate (MA) at both ends.

Contact Lens Monomers	Commercial Name	FDA Group	Ionic	Water Content [%]	Thickness τ [mm]	Radius *R* [mm]
Nelfilcon A PVA, FMA, PEG [14]	DAILIES Aquacomfort Plus	II	No	69 [14]	0.100 [14]	7.000 [14]
Omafilcon A HEMA, PC, EGDMA [14]	Proclear 1-Day	II	No	62 [14]	0.090 [14]	7.100 [14]
Etafilcon A HEMA, MAA [14]	1-Day Acuvue Moist	IV	Yes	58 [14]	0.080 [14,16]	7.150 [14,16]
Ocufilcon B HEMA, PVP, MAA [14]	Biomedics 1-Day	IV	Yes	53 [14]	0.070 [14]	7.100 [14]
Balafilcon A TRIS, NVP [37]	Pure Vision 2	V	Yes	36 [21]	0.090 [21]	6.250 [21]
Delefilcon A Not disclosed [14]	DAILIES TOTAL 1	V	No	33 [14]	0.090 [14]	7.050 [14]
Lotrafilcon A DMA, TRIS, Siloxane [38]	NIGHT & DAY	V	No	24 [21]	0.080 [21]	6.390 [21]
Lotrafilcon B DMA, TRIS, Siloxane [38]	O_2_ Optix	V	No	33 [21]	0.080 [21]	6.390 [21]
Narafilcon A HEMA, DMA, TEGDMA, Siloxane, MPDMS, PVP [14]	1-Day ACUVUE TruEye	V	No	46 [14]	0.090 [14]	7.100 [14]
SCL1 DMA, TRIS, EGDMA, MA-PDMS-MA [11]	-	V	-	38 [11]	0.084 [11]	6.005 [11]
SCL2 DMA, TRIS, EGDMA, MA-PDMS-MA [11]	-	V	-	30 [11]	0.082 [11]	5.970 [11]
SCL3 DMA, TRIS, EGDMA, MA-PDMS-MA [11]	-	V	-	25 [11]	0.081 [11]	5.880 [11]
Senofilcon A HEMA, DMA, Siloxane, TEGDMA, PVP, MPDMS [14]	Acuvue Oasys	V	No	20 [14,21]	0.084 [14,21]	7.150 [14,21]
Somofilcon A Not disclosed [14]	Clariti 1-Day	V	-	56 [14]	0.070 [14]	7.050 [14]

**Table 3:** Description of therapeutics and their respective properties.

Therapeutic	Molecular Mass [g/mol]	Density [g/cm^3]^	Molecular Volume [cm^3^/mol]	Charge
Atropine	289.37 [39]	1.20 [40]	241.14	Positive [41]
Bupivacaine	342.90 [39]	0.99 [42]	343.07	Positive [13]
Cysteamine	77.15 [39]	1.00 [40]	77.15	Positive [43]
Dorzolamide	324.44 [39]	1.60 [40]	202.78	Positive [44]
Fluconazole	306.27 [39]	1.50 [40]	204.18	Negative [45]
Flurbiprofen sodium	244.26 [39]	1.01 [40]	240.42	Negative [41]
Ketotifen fumarate	309.43 [39]	0.97 [46]	319.66	Positive [13]
Ketorolac tromethamine	255.27 [39]	1.33 [47]	191.93	Positive [32,48]
Levofloxacin	361.37 [39]	1.50 [40]	240.19	Positive [49]
Moxifloxacin	401.43 [39]	1.40 [40]	286.74	Positive [50]
Pirenzepine	351.40 [39]	1.30 [40]	270.31	Positive [41]
Red dye	650.60 [39]	1.00 [16]	650.60	Negative [51]
Tetracaine hydrochloride	200.80 [39]	1.13 [52]	178.00	Positive [13]
Timolol	432.50 [39]	1.20 [40]	360.40	Positive [53]

**Table 4:** Results of model validation. The input variables used to derive the estimated diffusion coefficient are reported, and the estimate ($D_{PDE}$) is compared to the value of *D* reported in the literature.

Lens Drug	Thickness *τ* [mm]	Radius *R* [mm]	Initial Mass Loaded *πR*^2^*τc*_*l*0_ [μg]	Estimated Value *D_PDE_* [$\frac{\mu m^{2}}{\mathrm{hr}}$]	Reported Value *D* [$\frac{\mu m^{2}}{\mathrm{hr}}$]
HEMA Cyclosporine	0.15	6.0	20.00	15.80	15.91 ± 1.90 [19]
HEMA Levofloxacin	0.30	6.0	24.52	2780	2700 [20]
Senofilcon A Red dye	0.084	7.150	47,800 ± 2700	mean: 25 range: 17–30	27.04 [24]
Etafilcon A Red dye	0.080	7.150	22,400 ± 2000	mean: 776 range: 607–800	813.60 [24]

**Table 5:** Kendall rank correlation coefficients, *r*, and *p*-values are reported to study correlations between $\log(D_{PDE}^{Mean})$, $\log(T_{50})$, and lens and therapeutics properties. The * symbol denotes statistically significant *p*-values <0.05.

	$\log(D_{\mathrm{PDE}}^{\mathrm{Mean}})$	Water Content	Thickness τ	Radius R	Molecular Density	Molecular Mass	Molecular Volume
Water content	r=0.34						
	p<0.001 *						
Thickness τ	r=0.15	r=0.22					
	p=0.165	p=0.043 *					
Radius *R*	r=−0.20	r=−0.08	r=−0.24				
	p=0.052	p=0.485	p=0.036 *				
Molecular density	r=0.23	r=0.20	r=−0.03	r=−0.06			
	p=0.020 *	p=0.055	p=0.764	p=0.557			
Molecular mass	r=0.04	r=0.03	r=−0.02	r=−0.16	r=0.06		
	p=0.700	p=0.749	p=0.840	p=0.143	p=0.557		
Molecular volume	r=−0.07	r=−0.03	r=−0.01	r=−0.15	r=−0.29	r=0.65	
	p=0.487	p=0.791	p=0.968	p=0.148	p=0.004 *	p<0.001 *	
$\log(T_{50})$	r=−0.78	r=−0.43	r=−0.09	r=0.20	r=−0.36	r=0.03	r=0.17
	p<0.001 *	p<0.001 *	p=0.399	p=0.058	p<0.001 *	p=0.751	p=0.089

**Table 6:** Mean, standard deviation (SD), and median of the estimated diffusion coefficient for the considered SH and CH lenses and the corresponding FDA groups. For normally distributed datasets (denoted by ^†^), *p*-values of the *t*-test for differences in means (≠, two-tailed) are reported, and when statistically significant (p<0.05), the *p*-values of the upper- or lower-tailed *t*-test are also reported. Mood’s median test results, with *p*-values, are reported for non-normally distributed datasets. The * symbol denotes statistically significant *p*-values <0.05.

Estimated Diffusion Coefficient $D_{\mathrm{PDE}}^{\mathrm{Mean}}$	Silicone Hydrogel (SH) Lenses (N = 36)	Conventional Hydrogel (CH) Lenses (N = 17)	
Mean ± SD $\left[\frac{\mu m^{2}}{h}\right]$ Median $\left[\frac{\mu m^{2}}{h}\right]$	$5.656\times 10^{3}$ ±$1.875\times 10^{4}$ $9.200\times 10^{2}$	$8.880\times 10^{3}$ $\pm 9.384\times 10^{3}$ $5.040\times 10^{3}$	
	**Group V** **(N = 36)**	**Group II ^†^** **(N = 8)**	**Group IV ^†^** **(N = 9)**
Mean		$1.593\times 10^{4}$	$2.613\times 10^{3}$
± SD $\left[\frac{\mu m^{2}}{h}\right]$	same as	$\pm 9.530\times 10^{4}$	$\pm 1.660\times 10^{3}$
	SH Lenses		
Median $\left[\frac{\mu m^{2}}{h}\right]$		1.231 $\times 10^{4}$	$2.289\times 10^{3}$
*t*-test, *p*-value	Group II ^†^ ≠ Group IV ^†^, p<0.001 * (Group II ^†^ > Group IV ^†^, p<0.001 *)		
Mood’s median test, *p*-value	SH ≠ CH, p<0.001 *		
	Group II ^†^ ≠ Group V, p=0.008 *		
	Group IV ^†^ ≠ Group V, p=0.008 *		

**Table 7:** Mean, standard deviation (SD), and median of the estimated diffusion coefficients ($D_{PDE}^{Mean}$) for the ionic, non-ionic, and unknown-ionicity lenses. Mood’s median test results for differences in medians (≠) are reported with the corresponding *p*-values. The * symbol denotes statistically significant *p*-values <0.05. The ^†^ symbol denotes the normally distributed dataset.

Estimated Diffusion Coefficient $D_{\mathrm{PDE}}^{\mathrm{Mean}}$	Ionic Lenses ^†^ (N = 10)	Non-Ionic Lenses (N = 38)	Unknown Ionicities Lenses (N = 5)
Mean	2.622 $\times \;10^{3}$	$8.277\times 10^{3}$	$1.632\times 10^{3}$
±	±	±	±
SD $\left[\frac{\mu m^{2}}{h}\right]$	$1.565\times 10^{3}$	$1.880\times 10^{4}$	$2.881\times 10^{3}$
Median $\left[\frac{\mu m^{2}}{h}\right]$	$2.494\times 10^{3}$	$1.319\times 10^{3}$	$3.000\times 10^{2}$
Mood’s median test, *p*-value	Ionic ^†^ ≠ Non-ionic, p=0.033 *		
	Ionic ^†^ ≠ Unknown ionicity, p=0.143		
	Non-ionic ≠ Unknown ionicity, p=0.170		

**Table 8:** Multiple linear regression model parameters for the two therapeutic molecular volume (Vol) groups, where *N* is the size of the dataset after removing the outliers. The outliers are lens–therapeutic combinations that were removed to ensure normality of residuals. Abbreviations are as follows: THCL: tetracaine hydrochloride; FBNA: flurbiprofen sodium; KTH: ketorolac tromethamine; Piren: pirenzepine; Moxi: moxifloxacin. The coefficient of determination with *p*-value and the regression coefficients ($a_{j}$) with 90% confidence intervals (${\mathrm{CI}}_{j}$) are reported. The * symbol denotes statistically significant *p*-values <0.05.

	75 < Vol < 275 [cm^3^/mol] (N = 26)	275 < Vol < 650 [cm^3^/mol] (N = 19)
Outliers: Lens, Therapeutic	Narafilcon A, KTH Etafilcon A, Piren Nelefilcon A, Piren Narafilcon A, Piren Narafilcon A, THCL Narafilcon A, FBNA	Delefilcon A, Moxi Narafilcon A, Moxi
Coefficient of determination, $R^{2}$, *p*-value	$R^{2}=0.64$, p<0.001 *	$R^{2}=0.84$, p<0.001 *
Regression coefficient, $a_{j}$, 90% Confidence interval (${\mathrm{CI}}_{j}$)	$a_{0}=-0.5378,$ ${\mathrm{CI}}_{0}=\left[-7.2270,6.1514\right]$ $a_{1}=-0.0413,$ ${\mathrm{CI}}_{1}=\left[-0.2055,0.1229\right]$ $a_{2}=0.5720,$ ${\mathrm{CI}}_{2}=\left[-4.4674,5.6114\right]$ $a_{3}=-0.0175,$ ${\mathrm{CI}}_{3}=\left[-0.141,0.1061\right]$	$a_{0}=18.2000,$ ${\mathrm{CI}}_{0}=\left[8.7262,27.6738\right]$ $a_{1}=-0.2003,$ ${\mathrm{CI}}_{1}=\left[-0.4162,0.0155\right]$ $a_{2}=-15.8068,$ ${\mathrm{CI}}_{2}=\left[-24.1743,-7.4393\right]$ $a_{3}=0.1527,$ ${\mathrm{CI}}_{3}=\left[-0.0270,0.3325\right]$

## Data Availability

All the data created in this study is reported in the tables of the manuscript and appendices.

## Abbreviations

The following abbreviations are used in this manuscript:

CH	Commercial hydrogel
SH	Silicone hydrogel
FDA	U.S. Food and Drug Administration
PBS	Phosphate-buffered saline
EGDMA	Ethylene glycol dimethacrylate
FMA	N-Formylmethyl acrylamide
PVP	Polyvinylpyrrolidone
NVP	N-vinylpyrrolidone
DMA	N,N-Dimethylacrylamide
PC	Phosphorylcholine
MPDMS	Monofunctional polydimethylsiloxane
TEGDMA	Tetraethleneglycol dimethacrylate
PVA	Polyvinyl alcohol
HEMA	Hydroxyethyl methacrylate
MAA	Methacrylic acid
TRIS	3-(trimethylsiloxy)silylpropyl methacrylate
PEG	Polyethylene glycol
FMA	Fluorinated methacrylate
MA-PDMS-MA	Polydimethylsiloxane (PDMS) long chains with methacrylate (MA) at both ends.
SD	Standard deviation
CI	Confidence interval
Cys	Cysteamine
THCL	Tetracaine hydrochloride
FBNA	Flurbiprofen sodium
KTH	Ketorolac tromethamine
Atro	Atropine
Fluco	Fluconazole
KF	Ketotifen fumarate
Dorzo	Dorzolamide
BUP	Bupivacaine
Piren	Pirenzepine
Levo	Levofloxacin
Moxi	Moxifloxacin
Timo	Timolol
RD	Red dye

## Appendix A. Justification of Sink Boundary Conditions

The mass of the therapeutic in the contact lens at time *t* ($m_{l}\left(t\right)$) is given by

(A1) $$m_{l}\left(t\right)=\pi R^{2}\int_{0}^{\tau }c_{l}\left(z,t\right)\;dz=v_{l}{\bar{c}}_{l}\left(t\right),$$

where $v_{l}$ denotes the contact lens volume and ${\bar{c}}_{l}\left(t\right)$ is the average therapeutic concentration in the contact lens. The contact lens volumes were estimated by assuming each contact lens is a cylinder with the radius given by *R* and height given by τ, both reported in Table 2, i.e., $v_{l}=\pi R^{2}\tau$. Therefore, the change in the contact lens therapeutic mass over time is computed as $m_{l}^{\prime }\left(t\right)=v_{l}{\bar{c}}_{l}^{\prime }\left(t\right)$.

The mass of the therapeutic in the vial at time *t* is given by

(A2) $$m_{v}\left(t\right)={\;\int \int \int \;}_{\Omega_{v}}c_{v}\;dV=v_{v}{\bar{c}}_{v}\left(t\right),$$

where $\Omega_{v}$ denotes the domain of the vial solution, $v_{v}$ is the volume of the solution in the vial, and ${\bar{c}}_{v}\left(t\right)$ is the average therapeutic concentration in the vial. Therefore, the rate of change of the vial’s therapeutic mass over time is computed as $m_{v}^{\prime }\left(t\right)=v_{v}{\bar{c}}_{v}^{\prime }\left(t\right)$.

Since the therapeutic mass was conserved in the experimental system and, initially, $c_{v}{|}_{t=0}=0$, the therapeutic mass in the vial is $m_{v}\left(t\right)=m_{l}\left(0\right)-m_{l}\left(t\right)$. Therefore, $m_{v}^{\prime }\left(t\right)=-m_{l}^{\prime }\left(t\right)$. Thus, the rate of change of the average therapeutic vial concentration is given by

(A3) $${\bar{c}}_{v}^{\prime }\left(t\right)=-\frac{v_{l}}{v_{v}}{\bar{c}}_{l}^{\prime }\left(t\right).$$

For all analyzed experiments, the ratio of the lens volume to the vial volume ($\frac{v_{l}}{v_{v}}$) was less than 0.7% (see Table A1 in Appendix B). Consequently, ${\bar{c}}_{v}^{\prime }\left(t\right)\approx 0$, so the average therapeutic vial concentration was approximately constant during the experiment, and because the therapeutic vial concentration was initially zero, $c_{v}\approx 0$.

Note that, using the notation introduced in this section, the cumulative release of the therapeutic from the contact lens defined in Equation (5) can be expressed as $m_{r}\left(t;D\right)=m_{l}\left(0\right)-m_{l}\left(t\right)$.

## Appendix B. Partition Coefficient Estimates

Table A1 reports all the numerical values, organized alphabetically by therapeutic, used to estimate the partition coefficient derived in Equation (1).

**Table A1:** Estimated values of the partition coefficient (see Equation (1)) for all considered lens–therapeutic combinations. The values of the therapeutic loading concentration and vial volume were extracted from the cited literature.

Therapeutic	Lens	Loading Conc $c_{v_{\mathrm{loading}}}$ [mg/mL]	Vial Volume $v_{v}$ [mL]	Lens vol $v_{l}=\pi R^{2}\tau$ [mL]	Partition Coeff *K*	$\frac{\mathrm{Lens}\mathrm{vol}}{\mathrm{Vial}\mathrm{vol}}$ $\frac{v_{l}}{v_{v}}$ [%]
Atropine	Delefilcon A [12]	10.00	4.0	0.014	0.0019	0.35
	Narafilcon A [12]	10.000	4.0	0.014	0.0021	0.36
	Ocufilcon B [12]	10.000	4.0	0.013	0.0076	0.32
	Etafilcon A [12]	10.000	4.0	0.013	0.0080	0.32
	Omafilcon A [12]	10.000	4.0	0.014	0.0052	0.36
	Nelfilcon A [12]	10.000	4.0	0.015	0.0020	0.38
Bupivacaine	Senofilcon A [13]	1.000	10.0	0.013	13.0376	0.13
	Narafilcon A [13]	1.000	10.0	0.014	19.5144	0.14
Cysteamine	Senofilcon A [29]	50.000	3.0	0.013	0.9192	0.45
	Narafilcon A [29]	50.000	3.0	0.014	0.9996	0.47
Dorzolamide	Senofilcon A [31]	0.800	3.5	0.013	12.0636	0.39
Fluconazole	Senofilcon A [21]	0.700	2.0	0.013	6.9860	0.67
	Lotrafilcon A [21]	0.700	2.0	0.012	6.9830	0.60
	Lotrafilcon B [21]	0.700	2.0	0.012	8.9742	0.60
	Delefilcon A [15]	1.000	4.8	0.014	10.5269	0.29
	Narafilcon A [15]	1.000	4.8	0.014	5.6290	0.30
	Somofilcon A [15]	1.000	4.8	0.013	6.3846	0.28
	Ocufilcon B [15]	1.000	4.8	0.013	14.9514	0.26
	Etafilcon A [15]	1.000	4.8	0.013	12.0924	0.27
	Omafilcon A [15]	1.000	4.8	0.014	9.0005	0.30
	Nelfilcon A [14]	1.000	4.8	0.015	3.0853	0.32
Fluobriprofen	Senofilcon A [32]	0.200	3.0	0.013	49.1691	0.45
sodium	Narafilcon A [32]	0.200	3.0	0.014	45.5571	0.47
Ketotifen	Senofilcon A [12]	0.300	10.0	0.013	44.1014	0.13
fumarate	SCL3 [11]	1.945	5.0	0.009	18.6872	0.18
	SCL2 [11]	1.945	5.0	0.009	23.2798	0.18
	SCL1 [11]	1.945	5.0	0.010	27.2340	0.19
	Narafilcon A [12]	0.300	10.0	0.014	44.1298	0.14
Ketorolac	Senofilcon A [12]	1.200	3.0	0.013	8.2319	0.45
tromethamine	Narafilcon A [12]	1.200	3.0	0.014	7.6045	0.48
Levofloxacin	Delefilcon A [11]	1.500	3.0	0.014	0.8624	0.47
Moxifloxacin	Delefilcon A [14]	1.000	4.8	0.014	1.6297	0.29
	Narafilcon A [14]	1.000	4.8	0.014	2.1655	0.30
	Somofilcon A [14]	1.000	4.8	0.013	2.6676	0.28
	Ocufilcon B [14]	1.000	4.8	0.013	17.1895	0.26
	Etafilcon A [14]	1.000	4.8	0.013	17.5985	0.27
	Omafilcon A [14]	1.000	4.8	0.014	9.7811	0.30
	Nelfilcon A [14]	1.000	4.8	0.015	2.5900	0.32
Pirenzepine	Delefilcon A [13]	1.000	4.0	0.014	0.0142	0.35
	Narafilcon A [13]	1.000	4.0	0.014	0.0161	0.36
	Ocufilcon B [13]	1.000	4.0	0.013	0.0253	0.32
	Etafilcon A [13]	1.000	4.0	0.013	0.0265	0.32
	Omafilcon A [13]	1.000	4.0	0.014	0.0372	0.36
	Nelfilcon A [13]	1.000	4.0	0.015	0.0182	0.38
Red dye	Senofilcon A [16]	1004.800	2.0	0.013	3.7044	0.67
	Etafilcon A [16]	1004.800	2.0	0.013	1.6533	0.64
Tetracaine	Senofilcon A [12]	0.200	10.0	0.013	41.4192	0.13
hydrochloride	Narafilcon A [12]	0.200	10.0	0.014	35.6946	0.14
Timolol	Senofilcon A [21]	0.800	2.0	0.013	8.8011	0.67
	Lotrafilcon A [21]	0.800	2.0	0.012	4.7859	0.60
	Lotrafilcon B [21]	0.800	2.0	0.012	7.4657	0.60
	Delefilcon A [18]	5.000	3.0	0.014	1.7667	0.47
	Balafilcon A [21]	0.800	2.0	0.011	14.8461	0.55

## Appendix C. Additional Results

Table A2 reports the mean and range of the initial therapeutic mass loaded into the contact lens; the mean ($D_{PDE}^{Mean}$) and range of the estimated diffusion coefficient ($D_{PDE}$); the predicted 50% release time of the therapeutic ($T_{50}$), assuming the diffusion coefficient of the therapeutic is $D_{PDE}^{Mean}$; and the relative average root mean square error ($\frac{\sqrt{E_{PDE}}}{v_{l}c_{l_{0}}n}$) of the parameter estimation algorithm, where *n* is the number of measurements.

**Table A2:** The value of the initial loaded therapeutic mass ($\pi R^{2}\tau c_{l_{0}}$) used to find the reported mean and range of the estimated diffusion coefficient ($D_{PDE}$). The predicted 50% release time ($T_{50}$) of the therapeutic, assuming the diffusivity of the therapeutic is given by the reported mean value ($D_{PDE}^{Mean}$). The relative average root mean square error ($\sqrt{E_{PDE}}/\left(v_{l}c_{l_{0}}n\right)$) of the parameter estimation algorithm is reported in the last column.

Therapeutic	Lens	Initial Loaded Therapeutic Mass $v_{l}c_{l_{0}}=\pi R^{2}\tau c_{l_{0}}$ [μg]	Estimated Diffusion Coefficient $D_{\mathrm{PDE}}$ $\left[\frac{\mu m^{2}}{\mathrm{hr}}\right]$	50% Release Time *T*_50_ [h]	Relative Error $\frac{\sqrt{E_{\mathrm{PDE}}}}{v_{l}c_{l_{0}}n}$
Atropine	Delefilcon A [12]	mean: 0.27 range: 0.25–0.28	mean: 16,400 range: 13,990–20,000	0.0210	1.33 $\times \;10^{-2}$
	Narafilcon A [12]	mean: 0.36 range: 0.35–0.38	mean: 6900 range: 6300–7500	0.0600	$6.33\times 10^{-3}$
	Ocufilcon B [12]	mean: 0.96 range: 0.94–0.99	mean: 4515 range: 4200–4800	0.0540	$4.41\times 10^{-3}$
	Etafilcon A [12]	mean: 1.03 range: 0.10–1.05	mean: 5808 range: 5400–6200	0.0522	$3.77\times 10^{-3}$
	Omafilcon A [12]	mean: 0.74 range: 0.71–0.77	mean: 14,680 range: 12,600–17,300	0.0202	$8.33\times 10^{-3}$
	Nelfilcon A [12]	mean: 0.31 range: 0.29–0.33	mean: 29,750 range: 27,900–31,700	0.0201	$1.06\times 10^{-2}$
Bupivacaine	Senofilcon A [13]	mean: 175.80 range: 170.33–181.27	mean: 80 range: 65–92	4.3100	$1.20\times 10^{-2}$
	Narafilcon A [13]	mean: 278 range: 232.59–320.96	mean: 117 range: 67–122	3.3800	$1.85\times 10^{-2}$
Cysteamine	Senofilcon A [29]	mean: 619.7 range: 569.7–669.7	mean: 10,200 range: 8300–12,700	0.0390	$1.81\times 10^{-2}$
	Narafilcon A [29]	mean: 712.4 range: 684.0–740.8	mean: 4310 range: 3880–4870	0.0923	$8.63\times 10^{-3}$
Dorzolamide	Senofilcon A [31]	mean: 122 range: 119.4–124.6	mean: 1500 range: 1400–1600	0.2615	$2.72\times 10^{-2}$
Fluconazole	Senofilcon A [15]	mean: 65.94 range: 64.48–67.39	mean: 690 range: 444–762	0.5000	$1.78\times 10^{-2}$
	Lotrafilcon A [15]	mean: 58.60 range: 57.29–59.64	mean: 920 range: 609–1122	0.3700	$5.12\times 10^{-3}$
	Lotrafilcon B [15]	mean: 75.13 range: 73.96–76.28	mean: 1130 range: 1055–1202	0.2700	$5.66\times 10^{-3}$
	Delefilcon A [15]	mean: 147.86 range: 142.19–153.53	mean: 1220 range: 1163–1813	0.0816	$1.95\times 10^{-2}$
	Narafilcon A [15]	mean: 80.19 range: 77.99–82.38	mean: 390 range: 367–606	0.0849	$1.88\times 10^{-2}$
	Somofilcon A [15]	mean: 86.09 range: 81.15–91.03	mean: 927 range: 357–2071	0.0219	$2.79\times 10^{-2}$
	Ocufilcon B [15]	mean: 189.33 range: 174.40–204.26	mean: 710 range: 430–936	0.0864	$1.83\times 10^{-2}$
	Etafilcon A [15]	mean: 155.29 range: 125.58–184.99	mean: 2288 range: 1830–3062	0.0212	$1.41\times 10^{-2}$
	Omafilcon A [15]	mean: 128.22 range: 107.09–149.35	mean: 5040 range: 4451–5587	0.0215	$1.31\times 10^{-2}$
	Nelfilcon A [15]	mean: 47.47 range: 32.68–62.26	mean: 4500 range: 4460–5587	0.0230	$2.58\times 10^{-2}$
Flubriprofen sodium	Senofilcon A [32]	mean: 132.7 range: 126.5–138.9	mean: 356 range: 337–385	0.9582	$1.39\times 10^{-2}$
	Narafilcon A [32]	mean: 129.8 range: 124.6–135	mean: 107.6 range: 107.1–108	3.6800	$4.33\times 10^{-2}$
Ketotifen fumarate	Senofilcon A [13]	mean: 178.40 range: 151.56–193.37	mean: 52 range: 45–61	6.6375	$1.19\times 10^{-2}$
	SCL3 [11]	mean: 319.62 range: 301.54–337.36	mean: 72 range: 70–95	3.8600	$1.92\times 10^{-2}$
	SCL2 [11]	mean: 415.52 range: 387.81–443.23	mean: 141 range: 108–150	2.3400	$1.53\times 10^{-2}$
	SCL1 [11]	mean: 503.81 range: 470.04–513.00	mean: 300 range: 250–422	1.1500	$2.43\times 10^{-2}$
	Narafilcon A [13]	mean: 188.60 range: 176.68–200.52	mean: 50 range: 35–95	7.9000	$3.27\times 10^{-2}$
Ketrorolac tromethamine	Senofilcon A [32]	mean: 133.2 range: 127.8–138.6	mean: 436 range: 434–440	0.7859	$1.23\times 10^{-2}$
	Narafilcon A [32]	mean: 130 range: 135–140	mean: 129 range: 126–132	3.0800	$1.12\times 10^{-2}$
Levofloxacin	Delefilcon A [18]	mean: 4500 range: 4408–4592	mean: 8900 range: 8700–10,405	0.2615	$8.93\times 10^{-3}$
Moxifloxacin	Delefilcon A [14]	mean: 22.89 range: 20.15–25.64	mean: 5700 range: 5190–5800	0.0703	$1.99\times 10^{-2}$
	Narafilcon A [14]	mean: 30.85 range: 27.54–34.16	mean: 320 range: 190–330	2.0337	$3.88\times 10^{-2}$
	Somofilcon A [14]	mean: 35.97 range: 34.77–37.18	mean: 6760 range: 6038–6800	0.0315	$7.45\times 10^{-3}$
	Ocufilcon B [14]	mean: 217.67 range: 214.04–221.31	mean: 1460 range: 1228–1550	0.1200	$3.63\times 10^{-3}$
	Etafilcon A [14]	mean: 226 range: 223.18–226.50	mean: 373 range: 300–401	0.1100	$5.30\times 10^{-3}$
	Omafilcon A [14]	mean: 139.34 range: 136.91–141.76	mean: 5880 range: 5540–5983	0.0400	$6.08\times 10^{-3}$
	Nelfilcon A [14]	mean: 39.85 range: 37.69–42.00	mean: 43,000 range: 32,555–44,000	0.0200	$2.30\times 10^{-2}$
Pirenzepine	Delefilcon A [12]	mean: 0.20 range: 0.19–0.21	mean: 11,600 range: 11,200–13,300	0.0315	$6.67\times 10^{-3}$
	Narafilcon A [12]	mean: 0.23 range: 0.22–0.24	mean: 151 range: 135–251	2.6300	$2.26\times 10^{-2}$
	Ocufilcon B [12]	mean: 0.32 range: 0.29–0.35	mean: 1890 range: 1800–2100	0.1400	$3.58\times 10^{-3}$
	Etafilcon A [12]	mean: 0.34 range: 0.30–0.38	mean: 2820 range: 2520–3100	0.8400	$3.86\times 10^{-3}$
	Omafilcon A [12]	mean: 0.53 range: 0.50–0.57	mean: 10,200 range: 1055–11,500	0.0517	$5.53\times 10^{-3}$
	Nelfilcon A [12]	mean: 0.28 range: 0.26–0.30	mean: 25,400 range: 22,380–29,300	0.0200	$5.92\times 10^{-3}$
Red dye	Senofilcon A [16]	mean: 47,800 range: 45,100–50,500	mean: 25 range: 17–30	13.8400	$1.78\times 10^{-2}$
	Etafilcon A [16]	mean: 22,400 range: 20,400–24,400	mean: 776 range: 607–800	0.3100	$7.47\times 10^{-3}$
Tetracaine hydrochloride	Senofilcon A [13]	mean: 111.70 range: 105.37–116.08	mean: 160 range: 110–200	2.1633	$1.69\times 10^{-2}$
	Narafilcon A [13]	mean: 101.70 range: 90.91–112.72	mean: 131 range: 124–149	3.0211	$9.35\times 10^{-3}$
Timolol	Senofilcon A [21]	mean: 94.94 range: 74.95–114.93	mean: 1250 range: 1240–1325	0.2820	$5.52\times 10^{-3}$
	Lotrafilcon A [21]	mean: 45.79 range: 25.79–65.78	mean: 1190 range: 1098–1826	0.2614	$1.23\times 10^{-2}$
	Lotrafilcon B [21]	mean: 71.43 range: 51.44–91.42	mean: 1760 range: 1609–3649	0.1764	$8.04\times 10^{-3}$
	Delefilcon A [18]	mean: 1500 range: 1489.93–1510.70	mean: 111,000 range: 8900–14,100	0.0458	$8.61\times 10^{-3}$
	Balafilcon A [21]	mean: 131.11 range: 127.51–134.70	mean: 2700 range: 2227–5316	0.1500	$4.10\times 10^{-2}$

## Appendix D. Outlier Removal Process

In this section, we detail the procedure used to remove the outliers in the multiple linear regression tool described in Section 3.3. Table A3 reports the results of the regression coefficient of determination ($R^{2}$), with the corresponding *p*-value, and of the Shapiro–Wilk test *p*-value of the regression residuals before and after removing the outliers for the two considered ranges of therapeutic molecular volumes.

**Table A3:** Multiple linear regression coefficient of determination ($R^{2}$) with the corresponding *p*-value and Shapiro–Wilk test *p*-value for the regression residuals before (pre) and after (post) removing outliers for the two therapeutic molecular volume (Vol) groups.

		75 < Vol < 275 [cm^3^/mol]	275 < Vol < 650 [cm^3^/mol]
Size of the sample *N*	Pre outlier removal	32	21
	Post outlier removal	26	19
Regression $R^{2}$ and *p*-value	pre-outlier removal	$R^{2}=0.30$, p=0.025	$R^{2}=0.67$, p<0.001
	post-outlier removal	$R^{2}=0.64$, p<0.001	$R^{2}=0.84$, p<0.001
Shapiro–Wilk test *p*-value	pre-outlier removal	p=0.024	p=0.047
	post-outlier removal	p=0.154	p=0.355

An iterative approach was used to remove the outliers listed in Table 8. A normal probability plot was created using the normplot MATLAB function to compare the distribution of the residuals to the normal distribution. The distance between the normal probability plot line, passing through the first and third quartiles of the data, and each data point was considered. The data point with maximum distance was selected as an outlier and removed. Once the outlier was removed, multiple linear regression analysis was performed using all the data except the outlier, and the normality of the residuals was tested using the Shapiro–Wilk test. If failed, i.e., if the *p*-value of the Shapiro–Wilk test was <0.05, then the next data point (taken from the residual dataset after removing the previously selected outlier) with the maximum distance to the normal probability plot line was selected as the next outlier and was removed. The process was repeated until the Shapiro–Wilk result was p≥0.05.
